# Socio-cultural barriers to physical activity participation among urban, educated postpartum women in Wuhan, China: a qualitative study

**DOI:** 10.3389/fpubh.2026.1748048

**Published:** 2026-03-31

**Authors:** Jiao Yuan, Jun Zeng, Haifei Chen

**Affiliations:** 1 School of Physical Education, Hubei University, Wuhan, China; 2 Hubei Leisure Sports Development Research Center, Wuhan, China; 3 Hubei Business College, Wuhan, China; 4 College of Physical Education, Yunnan Agricultural University, Kunming, China

**Keywords:** China, physical activity participation, postpartum women, qualitative research, socio-cultural barriers

## Abstract

**Introduction:**

Postpartum physical activity (PA) is crucial for physical and maternal health. However participation rates among Chinese women remain low. While common barriers like time constraints are documented globally, the underlying socio-cultural mechanisms specific to the Chinese context remain underexplored. This study investigates the unique socio-cultural factors hindering PA participation among urban, educated women in Wuhan, China, using an intersectional lens to examine how cultural, familial, and structural influences interact.

**Methods:**

A qualitative study was conducted, integrating an 18-month WeChat-based ethnographic observation of a postpartum care group in a community in Wuhan, along with semi-structured in-depth interviews with 17 women who were 6–18 months postpartum (*n* = 17). 12 were interviewed in-person and 5 by telephone.

**Results:**

Participation was low, 1/3 engaged in PA, commencing at 3 months (*n* = 2), 6 months (*n* = 1), or beyond 12 months (*n* = 2) postpartum. Analysis identified three interrelated socio-cultural barriers: (1) restrictions stemming from the traditional “Zuo yuezi” (confinement) custom and complex intergenerational dynamics; (2) constraints imposed by a gendered family division of labor and motherhood-centric parenting knowledge systems; and (3) inadequate public policies and social support structures.

**Discussion:**

These intertwined socio-cultural barriers form a self-reinforcing system that subordinates maternal self-care to collective family duties. The study reveals how these factors intersect to constrain women’s agency, offering a contextualized, intersectional analysis of postpartum health behavior in China. Addressing this requires multi-level interventions targeting cultural norms, equitable family labor, and institutional support to enable context-sensitive health promotion.

## 1 Introduction

Empirical studies have confirmed the positive effects of postpartum physical exercise on both physical and mental health (1–4), but participation rates among postpartum women remain low in both Chinese and Western contexts (5–7).

Research in Western settings generally categorizes barriers to postpartum physical activity into individual and environmental factors. Individual factors include income, number of children, negative family attitudes, and lack of childcare or exercise partners (5, 8, 9). Environmental factors encompass limited access to public transportation, concerns about community safety, and difficulties in finding affordable and suitable activities (10, 11, 12). However, these findings are not universally applicable. Women are a highly heterogeneous group, and postpartum physical activity participation is shaped by multi-layered and culturally embedded factors. In China, physical activity participation among postpartum women has received limited scholarly attention. Although a small number of studies have begun to systematically identify barriers to postpartum exercise, categorizing them as internal, interpersonal, and structural constraints (13) or as external and internal factors (14), these analyses often lack depth in uncovering the underlying social and cultural determinants, particularly within China’s unique socio-cultural milieu.

Similar postpartum confinement practices exist in Taiwan (Zuo yuezi), South Korea (Sanhujori), and Japan, though with variations (15, 16). However, no studies have applied intersectional frameworks to understand how multiple social positions shape experiences of these traditions. Feminist intersectionality offers a valuable lens for understanding such complex realities. As Brah and Phoenix (17) argue, intersectionality denotes “the complex, irreducible, varied and variable effects which ensue when multiple axes of differentiation—economic, political, cultural, psychic, subjective and experiential—intersect in historically specific contexts.” Initially formally articulated by Black legal scholar Kimberlé Crenshaw to articulate the compounded discrimination faced by Black women (18), intersectionality has since been widely adopted to analyze how categories such as gender, class, culture, and nation interact to shape lived experience (37). In this study, we adopt intersectionality as a research paradigm to illuminate the diversity and complexity of Chinese postpartum women’s experiences, refusing to treat “women” as a monolithic category. Against the backdrop of China’s declining birth rates and the national push for a fertility-friendly society. China’s shift from one-child policy (1980–2015) to encouraging two-child (2016) and three-child (2021) families reflects demographic anxieties (19). However, pro-natalist rhetoric lacks structural support: scarce public childcare, limited maternity leave, marginal father participation (20). This policy-practice gap is critical context for understanding why postpartum women shoulder disproportionate care burdens that preclude exercise. This is not only an issue concerning the physical and mental health of individual women but also a public health topic that should be considered in building a fertility-friendly society. Therefore, grounded in a socio-cultural perspective, this study utilizes intersectionality theory to examine how macro-level factors in China—including culture, customs, prevailing ideologies, traditional gender norms, and social development—collectively influence postpartum women’s engagement in physical activity. It focuses specifically on urban, educated women in Wuhan with access to healthcare and technology. Findings may not be transferable to rural, less-educated, or ethnic minority populations.

## 2 Materials and methods

This study adopted a qualitative research design, informed by feminist intersectionality theory, which integrated participatory observation. In qualitative inquiry, the researcher is the primary instrument for data collection, with understanding arising from direct engagement and lived experience (21). As a postpartum woman myself, the first author, occupied an “insider” role, which facilitated smoother access to the research community and enriched the interpretation. To maintain objectivity, during the interview, the first author deliberately suspended personal experiences, adopting a researcher’s stance in questioning and documentation to avoid leading the participants, and the involvement of two co-authors in study design and critical analysis further enhanced the rigor and balance of the research (Table 1).

**Table 1:** Demographic characteristics of the interview participants (*n* = 17).

Participant ID	Age (years)	Educational background	Occupation	Parity	Mode of delivery
Q1	32	Associate degree	Nurse	First child	Vaginal delivery
Q2	33	Bachelor’s degree	New media operations specialist	First child	Vaginal delivery
Q3	33	Associate degree	Accountant	First child	Cesarean section
Q4	32	Master’s degree	Librarian	First child	Cesarean section
Q5	33	Master’s degree	Training institution teacher	First child	Vaginal delivery
Q6	33	Bachelor’s degree	Lawyer	First child	Cesarean section
Q7	32	Bachelor’s degree	Community manager	Second child	Cesarean section
Q8	31	Bachelor’s degree	Primary school teacher	Second child	Cesarean section
Q9	31	Senior high school	Full-time mother	Second child	Cesarean section
Q10	31	Associate degree	Accountant	First child	Cesarean section
Q11	37	Junior high school	Beauty salon owner	First child	Cesarean section
Q12	33	Senior high school	Salesperson	First child	Vaginal delivery
Q13	33	Bachelor’s degree	Project inspector	First child	Vaginal delivery
Q14	28	Master’s degree	Counselor	First child	Cesarean section
Q15	33	Bachelor’s degree	Primary school teacher	Second child	Cesarean section
Q16	32	Associate degree	Assistant	First child	Vaginal delivery
Q17	33	Doctoral degree	University teacher	First child	Cesarean section

Sample reflects diversity but skews urban, educated: 70% (12/17) held associate degree or higher; all had smartphone access and healthcare—indicating middle-class status.

### 2.1 Research setting and data collection

Data collection unfolded in two phases.

Phase 1: Ethnographic Observation. The first author conducted 18 months of WeChat ethnographic observation within a postpartum care WeChat group affiliated with a community in Wuhan. This group comprised 432 women who had given birth between March and June 2024. The WeChat platform served as a vibrant forum for members to exchange information and experiences on a wide array of topics, including infant vaccination, parenting practices, postpartum recovery, weight management, and intergenerational dynamics between mothers-in-law and daughters-in-law. On August 1, 2024, the first author posted a message in the group to disclose her researcher identity and provide a study information sheet. WeChat chat records were collected by screening for and capturing messages containing the following keywords: “exercise/运动, weight loss/减肥, body shape/塑形”. From August 12, 2024, to January 31, 2026, a total of 183 messages from 26 distinct users were captured. These chat records were used for theme identification and triangulation with the interview data, Privacy was protected by removing names. However, this approach has limitations: all messages were posted by active group members, while many others remained silent, and public expressions within the group may differ from privately held beliefs (Table 2).

**Table 2:** Distribution of participants reporting each barrier theme (*n* = 17).

Core theme	Sub-theme	Participants (IDs)	Number of participants
Traditional–cultural tensions	Restrictions of “Zuo yuezi” practices	Q8, Q12, Q15	3
	Intergenerational conflict in grand parenting	Q4, Q5, Q11, Q17	4
Primacy of motherhood	Gendered division of family labor	Q5, Q7, Q9	3
	Motherhood constrained by knowledge systems	Q6, Q7, Q13	3
Institutional neglect	Insufficient public policy and social support	Q2, Q10, Q12	3

Participants may report multiple barriers; total *n* exceeds 17 across themes.

Phase 2: In-Depth Interviews. Building on the insights gained from this observational phase, the second phase involved semi-structured in-depth interviews with 17 women who were 6–18 months postpartum. Participants were initially recruited through an open invitation in the community’s postpartum care WeChat group, from which 10 members volunteered. Subsequently, a snowball sampling technique was employed, leveraging the initial participants’networks to recruit an additional 7 interviewees. All participants were 6–18 months postpartum. We sought to ensure diversity in participants’ occupation and educational background. To accommodate the participants’ childcare needs and scheduling constraints, 12 interviews were conducted in person, while 5 were held via telephone. Interview mode was participant-determined. Analysis revealed no systematic depth differences between phone (*n* = 5: Q6, Q10, Q13, Q16, Q17) and in-person interviews. Prior to each interview, all participants were verbally informed about the study’s purpose and signed the informed consent form. Each interview, lasting approximately 30–60 min, was based on a guide exploring core questions like, “Why did you not engage in physical exercise after giving birth?” and “Why were you able to engage in physical exercise during the postpartum period?” All participants were assured of anonymity (Table 3).

**Table 3:** Postpartum physical activity participation and primary barrier patterns by educational attainment (*n* = 17).

Educational attainment	*n*	PA participation, *n* (%)	Key barrier pattern
Bachelor’s degree or higher	10	4 (40%)	Time constraints + negotiation of intensive mothering ideology
Associate degree or lower	7	1 (14%)	Lack of accessible guidance + intensive care labor Dominance of cultural restrictions (e.g., Zuo yuezi)

WeChat ethnography received specific ethical approval (Research Ethics Committee for Humanities and Social Sciences, Hubei University, Approval No. HUBU-HSSEC-2025009, Date: 2025.11.3). Members were informed and provided implied consent through continued participation with explicit opt-out procedures.

### 2.2 Data analysis

The sample size was determined based on the principle of thematic saturation. After 12 interviews, preliminary three-theme structure emerged. We purposively sampled 5 additional participants for maximum variation (Q14-doctoral degree, Q9-full-time mother, Q11- age 37). No new themes emerged, confirming saturation, though intersectional nuances within themes became more apparent. Interviews transcribed verbatim in Mandarin, generating 51 pages. Braun and Clarke’s (22) thematic analysis was used: (1) All authors read 3 transcripts; first author read all repeatedly; (2) Line-by-line coding generated 127 initial codes; (3) Codes collated into 15 potential themes. (4) All authors reviewed; resolved discrepancy about “intergenerational conflict” placement through consensus; (5) Finalized 3 themes, 5 sub-themes through iterative discussion; (6) Selected representative quotations. The translation of the article was initially performed by the first author, with AI assistance for language editing and grammar correction. An English language professional then conducted back-translation to verify accuracy. Any discrepancies identified were resolved through discussion. During the process of naming thematic, a discrepancy arose regarding whether the sub-themes of “family gender division of labor” and “intergenerational conflict in grandparenting” should be categorized under the broader theme of “cultural tensions.” After repeated reading and recoding of the raw data, it was determined that “limitations of intergenerational support” should be classified under “Traditional-Cultural Tensions” while “gendered division of labor within the family” could be integrated into the theme of “Primacy of Motherhood”. Observational notes from the WeChat group were used to triangulate and contextualize the interview findings.

### 2.3 Trustworthiness and reflexivity

The first author’s insider status as a postpartum woman in Wuhan facilitated access but risked biasing interpretations toward educated, urban perspectives. To mitigate this, several reflexive strategies were employed. A reflexive journal was maintained to record assumptions. During interviews, neutral probes (e.g., “Help me understand…”) were used, and personal disclosure was delayed. In analysis, weekly debriefings with co-authors challenged interpretations, and the author’s self-interview was analysed alongside other data to identify divergences. Additional trustworthiness measures included prolonged 18-month engagement, triangulation of interview and WeChat data, peer debriefing with external researchers, and member reflections where five participants (Q4, Q5, Q8, Q11, Q12) confirmed that the researcher’s description of their “postpartum exercise barriers” (e.g., family caregiving burdens, constraints from traditional cultural tensions) was consistent with their actual experiences.

## 3 Results

Among the 17 interviewees, only 5 reported participating in physical activities during the postpartum period, commencing at 3 months (*n* = 2), 6 months (*n* = 1), and after 12 months (*n* = 2) postpartum. Through collaborative thematic analysis, three core themes were identified: traditional-cultural tensions, motherhood prioritization, and institutional neglect.

### 3.1 Traditional-cultural tensions

#### 3.1.1 Restrictions of “Zuo yuezi”custom

“Zuo yuezi” is a traditional postpartum health practice prevalent in China, centred on adherence to various “confinement taboos” encompassing dietary, behavioural, and environmental restrictions (23). Rooted in Traditional Chinese Medicine, these practices aim to help mothers recover vital energy and blood loss through rest. Behavioural taboos include prohibitions on going outdoors, exposure to wind, washing hair, bathing, and emphasize bed rest and minimized physical movement—including household chores and even holding the infant. Environmental taboos require staying in enclosed spaces, avoiding wind, wearing long sleeves, pants, socks, and head coverings regardless of season. These restrictions fundamentally conflict with physical activity requirements. Although some taboos like hair washing are no longer strictly followed, the underlying belief system remains influential. Women often fear that non-compliance will lead to “yuezi bing” (confinement sickness) (24), a concern frequently reinforced by older female relatives who supervise based on their own experiences.

*The older generation of women, like mother-in-law and mother, usually say not to look at the phone, not to wash your hair, not to be exposed to the wind, wear a hat, wear long sleeves and long pants, sleep more in bed, and do not sit on the sofa, otherwise you will get “yuezi bing” later, like insomnia and headaches. (Q12).*

Most interviewees acknowledged the perceived rationality of “rest and recuperation,” believing extended bed rest facilitates physical recovery. All participants explicitly excluded physical activity during the traditional “confinement month,” with many extending this rest period and only considering exercise 3 months postpartum or later.

*I started exercising three months after giving birth. After I had the baby, my mother-in-law and my husband were the ones primarily taking care of the baby. For the first two months, I mostly just ate and slept. I needed a lot of sleep during that initial recovery phase. After sleeping for over two months, I found myself just lying around every day, feeling utterly bored. Therefore, I wanted to find something to do, and that’s why I started climbing stairs. (Q15).*

Another respondent provided evidence from her personal experience about not exercising too early. She said: *I had a C-section and only began regular exercise six months postpartum. Actually, I had attempted to exercise around four months after delivery. After one circuit training session that included both upper and lower body exercises, I experienced vaginal bleeding. It was likely triggered by the lower body strength training, particularly bodyweight squats. I’m not sure whether it was clotted blood or another kind of bleeding problem. But it was clearly uncomfortable. The bleeding stopped after I discontinued the exercises. Later, I did not resume training until after six months postpartum. (Q8).*

Some women in the XX community postpartum care WeChat group even expressed that vigorous-intensity physical activity, like jumping should only be chosen 1 year after childbirth. The reason is that the internal organs are displaced during pregnancy and need a considerable amount of time to return to their positions after delivery, making running and jumping very damaging. Consequently, they recommend opting only for gentle forms of physical activity until sufficient time has passed, such as walking, diaphragmatic breathing, and low-impact aerobics that avoid running and jumping.

#### 3.1.2 Intergenerational conflict in grandparenting

Unlike Western contexts where grandparental childcare typically occurs in exceptional circumstances, such as imprisonment or having a child out of wedlock (25), In China, caring for grandchildren is considered a traditional intergenerational ethic, making collaborative parenting a common practice. Grandparents often serve as “helpers,” undertaking substantial childcare and household responsibilities (26). Influenced by patriarchal traditions and patrilocal residence patterns, paternal grandparents—particularly mothers-in-law—typically provide this support, complicating intergenerational relationships. Differences in experience, culture, and beliefs between generations often lead to conflicts in labor division and parenting approaches. Some women reported that insufficient or undesirable intergenerational support forced them to devote extensive time to infant care and housework, leaving no opportunity for physical activity.

*My mother-in-law does not take care of the baby much, and when she does, she’s quite careless. She might stay over to sleep with the baby occasionally, but overall, I really have not had supportive in-laws. Earlier, I said my mother-in-law was lazy and did not want to do too much housework, but her son (my husband) kept saying that is* because *her health was getting worse and worse. Later, I stopped doing all those housework chores too, and only then did he realize his mom is actually just lazy. (Q11).*

Consequently, Q11 juggled work and childcare alone. Her daily routine unfolds as follows: she goes to the shop at around 9 a.m. to organize work-related affairs then after the baby finishes drinking milk, they have 2 h of outdoor activity, and then she has to return home to make lunch for the baby. After lunch, they play for a while, she puts the baby down for a nap at approximately 2 p.m. In the afternoon, she goes back to the shop to work for two or 3 h, then gets off work at 6 p.m. and heads home to cook dinner. After dinner, she spends one to 2 h playing with the baby, and around 9 p.m., she bathes the baby and puts him to bed. Q11’s husband, being occupied with work, can only take the child out for a brief outing after dinner. Regarding postnatal exercise, she has never been fond of vigorous-intensity physical activities. On the contrary, she is willing to try low-intensity physical activities such as yoga, but after a full day of being busy, she just wants to lie down and play with her phone—she simply has no time to practice yoga at all. Even during the interview she had to bring her child along. This account illustrates a common dilemma faced by many postpartum women.

*I really want to exercise after giving birth, but it’s just too difficult. For one thing, I do not have the time to exercise. For another, there’s no one who can genuinely help me with childcare. My mother-in-law is quite careless and also likes to smoke. My mother is occupied with her business, and my husband is busy with work too. That leaves me as the primary – and essentially sole – caregiver. It’s hard enough for me to even step out for a short walk by myself, let alone dedicate time specifically for physical activity participation. (Q5).*

The primary reason why Q4 only began exercising 1 year postpartum was the insufficient intergenerational support from her mother-in-law in the early stage. While helping to care for her child, her mother-in-law also needed to go to her elder son’s home in a nearby residential complex to care for her two older grandsons, even though her child was much younger and in greater need of care. This meant Q4 often had to bear most of the childcare responsibilities and housework. After her baby was over a year old, she adjusted her childcare strategy. On weekdays, she entrusted her child to her parents for care, and on weekends, she would either bring the child back or go to her parents’ home to care for the child together. By switching the intergenerational cooperative parenting model, she was able to secure stable, freely disposable time after work on weekdays to play tennis and table tennis with colleagues or junior schoolmates. Sometimes, she would also go for a slow jog in the park with her husband.

Furthermore, a subtle competitive dynamic can form between grandparents and young parents over forming an intimate bond with the child (27). This competition, especially with the paternal grandmother, can make women more willing to invest their time in infant care rather than using it for physical activity.

*During my maternity leave, my mother-in-law came to help take care of the baby, so I did have time to exercise, but I still did not do it. One reason is that I do not get along well with my mother-in-law. I believe that when a mother-in-law comes to help with the baby, it should be to assist with some housework and occasionally help with the baby, which would leave the mother with more time and energy to spend with the baby and build a better parent–child relationship. But in reality, my mother-in-law did not do what I wanted, or she did the opposite. She always wanted to takes care of the baby, as if vying for the baby with me, but she did not want to do much housework. (Q17).*

### 3.2 Primacy of motherhood

#### 3.2.1 Family gender division of labor

Under the traditional gendered division of labor where “the man is the breadwinner and the woman is the homemaker,” fathers typically provide financial support while mothers manage household affairs and childcare. Although shared parenting has become common in Western dual-earner families (28), China maintains a distinct gendered division, particularly during infancy, where mothers shoulder more childcare with relatively insufficient father involvement.

Influenced by the traditional gendered division of labor in the family, postpartum women tend to prioritize their children’s needs, placing themselves in a secondary position and organizing their daily lives around the children. Driven by the overwhelming childcare pressures following the birth of her second child, Q9, for instance, was compelled to exit the labor force and assume the role of a full-time mother. In the case of Q5, due to her husband’s long commute and frequent overtime work, they arranged for him to rent an apartment near his company, residing there on weekdays and returning home only on weekends. This arrangement resulted in the father’s complete absence from childcare from Monday to Friday, forcing Q5 to shoulder the full burden of parenting alone. She had to sacrifice her daily lunch break to return home and care for their baby, and sometimes even resorted to covertly leaving work (sneaking away) to manage her caregiving duties. Similarly, in Q7’s family, the “father earns money, mother provides care” model of gendered division of labor is still followed. Her husband primarily focuses on providing economic support for the family, with minimal participation in daily caregiving activities. Consequently, her husband is very free and can go on trips whenever he wants. “During one video call, I noticed while it was already dark here, his location remained in daylight. I later found out he was traveling in Xinjiang.” In contrast, Q7’s freedom is constrained by the children. Even with support from both sets of parents, she must return home directly after work, with no opportunity for personal time outside the home.

Furthermore, because grandparents are influenced and shaped by the “man as breadwinner, woman as homemaker” gender division, they often extend this tradition to their support of their children’s parenting. Under intergenerational childcare support, fathers tend to reduce both the relative and absolute time they spend on childcare, and are less likely to take on the role of nighttime caregiver (29). Particularly when paternal grandparents are involved in co-parenting, childcare responsibilities fall primarily on the mother and the grandparents. Many women complain that “the mother-in-law’s help with the baby only reduces the father’s burden, the mother still has to do all her work.” For working women, the lack of paternal involvement exacerbates the mother’s “second shift” burden, severely squeezing the time and energy that could otherwise be used for physical activity.

#### 3.2.2 The bondage of motherhood by knowledge systems

While the current scientific knowledge system contributes to healthy child-rearing, it simultaneously and inadvertently intensifies the burdens of motherhood. The knowledge system of “scientific parenting” emphasizes the irreplaceable primary position of the mother in a child’s cognitive and social development, to some extent binding the mother to the child in a primary caregiving relationship (30). Hospitals begin imparting knowledge on fetal and infant care to expectant mothers during the prenatal period. After childbirth, hospitals emphasize the benefits of breastfeeding, with maternity nurses guiding and assisting new mothers in initiating lactation as early as possible. When initial attempts are unsuccessful, medical staff implement various measures to stimulate milk production. Additionally, the walls of the maternity ward are covered with educational materials on breastfeeding, allowing mothers to absorb this information during their postpartum check-up intervals. Fundamentally, breastfeeding reinforces the mother’s irreplaceable role in relation to her child. The scientifically established notion that breastfeeding is superior to formula feeding compels many postpartum mothers to consider postponing exercise for weight loss until after weaning. This is because effective weight reduction typically requires a combination of physical activity and dietary control, yet restricting food intake raises concerns about diminishing the nutritional quality of breast milk.

Likewise, both psychology and neuroscience underscore the significance of early mother-infant attachment and the mother’s role in shaping a child’s cognitive and intellectual development (31). These scientific knowledge systems are accepted and internalized by new mothers through channels such as hospitals, parenting experts, and the media. Especially under the influence of factors like the cost of child-rearing and life pressures, the “post-90s”generation, as the main force of childbearing, generally shows a preference for having only one child. For mothers with just one child, this creates a “one-time motherhood experience” further intensifying their focus on scientifically-grounded parenting approaches. As they transition into the role of caregivers, these new mothers simultaneously become active learners of childcare knowledge. They proactively acquire modern parenting information through multiple channels: online research, including following pediatricians on platforms like TikTok and Xiaohongshu, consulting parenting literature, and peer consultations. This enables them to comprehensively understand and implement knowledge spanning infant growth milestones, nutritional requirements, early childhood education, outdoor activities, and parent–child games.

*Actually, I really want to practice yoga. After giving birth a year ago, I feel my physical condition has deteriorated significantly. That’s why I’m considering starting with yoga. However, I feel that taking out two days a week for it is still very difficult. From Monday to Friday, I have to work. On weekends, I feel my mother and father in-laws deserve a break from childcare. Personally, I also want to spend more time with my daughter. There just is not enough time for exercise. There are so many things to do every week, like wanting to visit my parents or arrange a parent–child playdate with friend, and I’m also preparing to take her to early education classes in September. (Q7).*

*Even though my mother-in-law helps with childcare, it’s still better if I can take care of him myself. I want to spend more time with him. My baby is quite clingy with me. I usually take him for outdoor activities in the community after I get off work in the afternoon. (Q6).*

These knowledge systems reinforce intensive motherhood ideology, advocating that mothers provide not only physical care but also substantial time and emotional commitment, compelling postpartum women to dedicate limited non-work time to high-quality parenting rather than self-care, When their own needs conflict with those of their child, they prioritize the child, fearing they might miss the critical period requiring maternal presence.

### 3.3 Institutional neglect: insufficiencies in public policy and social support systems

Although the China’s Postpartum Healthcare Services Guideline indicates that postpartum exercise can include various forms based on physical condition and personal preference such as diaphragmatic breathing, supine exercises, strength training, aerobic exercise, yoga, and pelvic floor muscle exercises (Kegel exercises). The guideline recommends that in the first 4 weeks postpartum, breathing function and strength training should be conducted progressively while also improving cardiopulmonary function. Between 4 and 6 weeks postpartum, regular aerobic exercise may be initiated, with intensity gradually increased according to physical recovery and individual tolerance. For women with other comorbid medical conditions, exercise plans may be appropriately adjusted based on medical advice. Lactating mothers should be advised to breastfeed before exercising to avoid discomfort caused by breast engorgement during physical activity (32). However, in practice, doctor’s advice on postpartum exercise during check-ups is generally limited to diastasis recti and pelvic floor muscle laxity. Doctors and nurses will advise mothers with these conditions to practice diaphragmatic breathing and Kegel exercises at home. Beyond this, few doctors recommend restoring health through exercise. For postpartum women, especially those with lower levels of education, they are unable to obtain free, scientific advice on postpartum exercise from authoritative institutions and thus lack knowledge about postpartum exercise recovery.

*The postpartum exercise programs offered by many confinement centers are largely targeted at mid-to-high-end clients. No one has ever explained to me the potential adverse effects of not exercising after childbirth, but I can clearly feel that my hips have become much wider. (Q12).*

*I had not considered the need for exercise recovery after childbirth, and my postpartum check-up did not detect any issues either. (Q10).*

*The doctor did not proactively recommend any exercise regimen. It was only during my postpartum check-up that I asked if I could exercise, and the doctor told me to try to avoid high-impact activities like running and jumping. (Q2).*

Meanwhile, Postpartum women experience a distinct period of physical and mental health, which requires their exercise regimens to adhere to specialized and individualized principles, avoiding any arbitrary or indiscriminate approach. However, as relevant public service sectors have not integrated postpartum exercise and healthcare into their service frameworks, this has resulted in an institutional supply gap regarding both exercise facilities and professional guidance for postpartum women.

Beyond lacking targeted policies supporting physical activity, complementary support systems are severely deficient. Since market economy implementation, withdrawn public childcare resources have returned child-rearing responsibility to families, subjecting women to the dual pressures of “family and work,” and increasing burdens (33). Furthermore, brief paternity leave and inadequate parental leave implementation have exacerbated women’s caregiving responsibilities.

### 3.4 Intersectional variations in barrier experiences

While traditional-cultural tensions, motherhood prioritization, and institutional neglect represent the primary barriers to physical activity (PA), significant variations in how these barriers were experienced emerged within the cohort, closely associated with differences in educational attainment and occupational patterns.

Educational differences significantly shaped how women navigated barriers. Among the respondents with a bachelor’s degree or higher (*n* = 10), 4 engaged in regular PA. This group demonstrated initiative in researching exercise protocols and the ability to use evidence in family negotiations. In contrast, among respondents with an associate degree or lower (*n* = 7), only 1 participated in PA. This group commonly reported a lack of professional guidance and limited awareness, exemplified by Q12’s statement: “No one has ever explained to me the potential adverse effects of not exercising after childbirth…”.

Occupational patterns further structured these constraints. Professionals with maternity leave (Q2, Q4, Q8, Q14) benefited from structured recovery time and institutional support. Conversely, self-employed or full-time mothers (Q11, Q12) lacked both protected time and stable income, resulting in having neither the time nor financial means for PA. Analysis of participant data indicated that factors such as parity and delivery mode were not decisive determinants of PA engagement in this sample.

Thus, the experience of socio-cultural barriers was not uniform. Higher educational and occupational capital provided resources—knowledge, negotiation leverage, and time security—that enabled some women to partially overcome or navigate these constraints. Lower levels of such capital left other women more fully subjected to them, facing barriers that were less negotiable and more absolute.

### 3.5 Enabling factors: women who successfully engaged in PA

Five participants (Q2, Q4, Q8, Q14, Q15) sustained regular physical activity. Their cases reveal enabling factors that offset the prevailing barriers: educational capital, strategic family resource restructuring, supportive care arrangements, and economic stability derived from professional employment. 4 possessed at least an associate degree. This educational background correlated with proactive health awareness. As Q2 noted, concern over significant postpartum weight gain motivated her to exercise. They utilized evidence-based knowledge to justify self-care within family settings. Strategically, some reconfigured childcare support. Q4 transferred primary care from her mother-in-law to her own mother, securing predictable time for tennis and jogging. Others benefited from existing supportive arrangements: Q15’s mother-in-law and husband assumed primary infant care, affording her discretionary time. Economically, professional employment provided structured maternity leave, ensuring income continuity and a legitimate recovery period, which facilitated planning. In one instance (Q8), economic resources allowed for purchasing childcare support.

Collectively, these factors illustrate how intersecting educational and economic capital enabled access to information, negotiation leverage within family hierarchies, and the capacity to secure time—whether through kin networks or market solutions—thus creating pathways to physical activity despite pervasive socio-cultural constraints.

## 4 Discussion

This study applies an intersectional feminist lens to analyze the socio-cultural barriers to physical activity participation among postpartum women in China, contributing a contextualized theoretical perspective from China to global debates on gender and maternal health. Our findings converge with and diverge from global maternal health literature. They confirm, like studies in Western contexts, that postpartum women universally face constraints such as time scarcity (36) and a tendency to prioritize caregiving over self-care (34). Yet, the socio-cultural mechanisms underlying these constraints are distinctly local. A telling contrast emerges in intergenerational care: while studies in African contexts [e.g., (35)] highlight support and constraints within extended family networks, our study reveals a specific dyadic tension in China, often centred on the maternal grandmother or mother-in-law within patrilocal norms. This comparison underscores that while the challenge of navigating postpartum recovery is global, its specific architecture—in China, shaped by a rigid gendered division of labor, intergenerational dynamics, and systemic support gaps—is uniquely local. Consequently, this analysis underscores the necessity of moving beyond generalized frameworks to capture the intersectional pathways that uniquely shape maternal health behavior in specific cultural settings.

First, traditional practices such as “Zuo yuezi”and intergenerational co-parenting, while culturally valued, also pose specific constraints. While both “Zuo yuezi” and intergenerational co-parenting are beneficial for postpartum recovery, they may simultaneously function as obstacles to physical exercise for new mothers. Meanwhile, “Zuo yuezi” acts as a form of gendered bodily discipline, directly influences the timing and feasibility of postpartum physical activity. Its “behavioural taboos”—such as prohibitions against going outdoors, exposure to wind, and even minimal physical movement—directly contradict the basic requirements of physical activity. Consequently, most interviewees excluded physical activity entirely during the confinement period, with many delaying exercise consideration until at least 3 months postpartum—thereby missing the critical window for early postpartum recovery. At the same time, intergenerational parenting often embodies contested care ethics and power dynamics between the younger and grandparent generations regarding parenting styles and the division of housework. These tensions can result in mothers lacking sufficient time for physical activity or being reluctant to allocate time to self-care.

Second, In China, women’s growing participation in the public sphere exists alongside the persistent patriarchal gender division of labor—men as breadwinners, women as homemakers—in the private familial domain. This dynamic, coupled with the authoritative, scientized discourse of mothering that reinforces the mother’s primary caregiving role, collectively subjects postpartum women to the hegemony of intensive motherhood ideology, This reinforces a mindset in which mothers prioritize their children’s needs over their own, leading them to sacrifice physical activity for the demand of “high-quality” morally charged parenting. As illustrated by participants such as Q7, even when women express a desire to exercise, they often abandon such plans to spend more time with their children or to support grandparents in caregiving.

Third, the state’s relative absence in providing care creates a structural gap that fails to counterbalance these cultural and familial barriers. Although China’s Postpartum Healthcare Services Guideline outlines various forms of postpartum exercise, medical guidance during check-ups is often limited to specific issues such as diastasis recti and pelvic floor muscle laxity, with little comprehensive advice on physical activity for overall recovery. Publicly accessible, affordable, and tailored postpartum exercise programmes—such as community-based classes—remain scarce. Moreover, the privatization of childcare shifts child-rearing responsibility entirely onto families, intensifying the work–family conflict for postpartum women, especially those who have returned to employment. Even when women attempt to adjust childcare strategies privately—as in the case of Q4, who entrusted her child to her parents to free up time—they rely on fragile, kin-based arrangements rather than systemic support, highlighting the absence of institutionalized rights to maternal health and bodily autonomy.

Collectively, these findings show that low physical activity is not merely an individual choice but a structural outcome of intersecting cultural, gendered, and institutional forces. A closer intersectional examination reveals that the impact of these forces is not uniform but is significantly mediated by socio-economic resources, particularly education. Women with bachelor’s degree (40% PA participation) demonstrated greater ability to navigate these constraints compared to those without one(14% participation), underscoring how educational capital fundamentally shapes the capacity to negotiate cultural norms, familial expectations, and institutional gaps. Furthermore, rather than only removing barriers, interventions should seek to strengthen the enabling factors identified among active participants: enhancing health literacy, facilitating strategic family support restructuring, and creating accessible childcare options independent of intergenerational negotiations.

This insight contextualizes the distinct barriers in China and underscores the need for interventions that target these underlying structural and ideological formations. These findings carry immediate implications for physiotherapy practice: (1) Culturally-sensitive counseling: frame exercise as “gentle recovery movement” and acknowledge Zuo yuezi; (2) Family-centred care: include mothers-in-law and partners; (3) Optimal timing: prioritize the 3–6 month postpartum window; (4) Accessible formats: develop short, home-based, infant-inclusive programs; (5) Intersectional assessment: tailor programs by screening education, family support, and work demands.

## 5 Conclusion

This study highlights the need for culturally-grounded approaches to maternal health, as seen in the distinct intergenerational dynamics within Chinese families compared to other settings. The findings point to multi-level interventions. At the institutional level, action should include: (1) integrating structured exercise counseling into standard 6-week and 3-month postpartum check-ups; (2) piloting community-based exercise programmes with on-site childcare in Wuhan neighborhoods; and (3) policy reforms such as extending paternity leave to a minimum of 30 days, subsidising childcare for infants aged 6–18 months, and offering flexible return-to-work options. At the cultural level, efforts should focus on: (1) developing evidence-based materials that reconcile Zuo yuezi traditions with modern exercise science; (2) collaborating with TCM practitioners to legitimise this integrated approach; and (3) conducting intergenerational workshops to update grandmothers’ knowledge. At the family level, initiatives should involve: (1) prenatal sessions on postpartum labor division; (2) mandatory father participation in antenatal classes; and (3) postnatal support groups for fathers.

Implementation should be phased: revising protocols and developing materials (Phase 1: 0–6 months), piloting community programmes (Phase 2: 6–18 months), and scaling successful models citywide (Phase 3: 18+ months). This requires collaboration across public health, maternal-child health centres, community organisations, and women’s federations. Crucially, interventions must strengthen enablers—such as health literacy, father involvement, and accessible childcare—rather than solely addressing barriers.

These conclusions are specific to the urban, middle-class context of this study. Future research must validate and adapt these insights for rural, ethnic minority, and lower-income populations across China.

### 5.1 Research limitations

This study has several limitations that guide future research. First, its focus on urban, educated women in Wuhan limits generalizability to rural, ethnic minority, or lower-income populations. Second, while the sample varied in education and occupation, the analysis prioritized identifying common systemic barriers over conducting in-depth intersectional subgroup comparisons. Third, the cross-sectional design cannot capture how barriers evolve across postpartum stages. Future studies should expand to more diverse populations, employ longitudinal designs to track temporal dynamics, investigate the experiences of women with postpartum complications, and conduct focused intersectional analyses (e.g., by education, occupation, parity) and cross-provincial or East Asian contexts (Taiwan, Korea, Japan) comparative research. Piloting culturally-adapted interventions and incorporating fathers’ perspectives are also crucial next steps.

## Data Availability

The raw data supporting the conclusions of this article will be made available by the authors, without undue reservation.
